# The HALF-IT score: proposing a simplified mechanical thrombectomy triage tool for basilar artery occlusion

**DOI:** 10.3389/fneur.2026.1915436

**Published:** 2026-10-14

**Authors:** Ann Marie Jones, Zain Tariq, Yazan Ashouri, Amit Chaudhari, Mohammad AlMajali, Mohamad Ezzeldin, Mustafa Abdul Kareem, Ahmad R. Awwad, Gregory Wong, Amir M. Molaie, Borja Sanz Cuesta, Jeffrey L. Saver, Kaustubh Limaye, Osama O. Zaidat

**Affiliations:** 1 Department of Vascular and Endovascular Neurology, Indiana University School of Medicine, Indianapolis, IN, United States; 2 Department of Neuroscience, Bon Secours Mercy Health St. Vincent Hospital, Toledo, OH, United States; 3 Department of Neuroendovascular Surgery, Ochsner Clinic Foundation Lafayette General Hospital, Lafayette, LA, United States; 4 Department of Neuroendovascular Surgery, HCA Houston Healthcare Kingwood, Kingwood, TX, United States; 5 Department of Vascular and Endovascular Neurology, University of California, Los Angeles, CA, United States

**Keywords:** basilar artery occlusion, endovascular thrombectomy, ischemic stroke, neuroimaging, triaging tool

## Abstract

**Background:**

Basilar artery occlusion (BAO) is a devastating subtype of stroke with poor prognosis. Current patient selection tools for endovascular thrombectomy (EVT) are multi-tiered with interrater variability and limited validation studies. The HALF-IT score is a novel, simplified, dichotomized triaging tool focusing solely on the brainstem level. This study investigated the utility of the HALF-IT score to predict functional outcomes of EVT in patients with BAO.

**Methods:**

We conducted a multicenter, retrospective, cohort study including patients with acute BAO presenting to four comprehensive stroke centers between 2021–2025. Acute infarction of ≥50% of any brainstem section on any axial or coronal slice of MRI was considered HALF-IT positive, whereas < 50% was considered HALF-IT negative. The primary efficacy outcome was a favorable functional outcome, defined as a modified Rankin Scale (mRS) score of 0–3 at 90 days; functional independence (mRS 0–2) was analyzed as a secondary outcome. Reperfusion was graded with the modified Thrombolysis in Cerebral Infarction (mTICI) scale. Primary safety endpoints included symptomatic intracranial hemorrhage at 24 h and all-cause mortality at 90 days. Multivariable logistic regression was performed to identify independent predictors of the primary efficacy outcome.

**Results:**

37 patients (23 HALF-IT negative; 14 HALF-IT positive) met the inclusion criteria. The rate of 90-day mRS 0–3 was significantly lower in the HALF-IT positive compared with HALF-IT negative group [14.3% vs. 68.2%, OR 0.08 (0.01–0.45), *p* = 0.004]. HALF-IT positive status remained associated with a lower rate of 90-day mRS 0–3 after adjusting for age, initial NIHSS, onset-to-reperfusion time, intravenous thrombolysis administration complete reperfusion (final mTICI 3) [adjusted OR 0.02, 95% CI 0–0.84, *p* = 0.02]. Safety events were numerically higher in the HALF-IT positive group but the difference did not reach statistical significance. The two groups differed substantially at baseline in age, presenting NIHSS, and intravenous thrombolysis exposure, whereas stroke etiology (*p* = 0.28) and level of basilar occlusion (*p* = 0.63) were similarly distributed.

**Conclusion:**

In this small, retrospective, hypothesis-generating cohort, HALF-IT positivity was associated with worse 90-day functional outcomes after EVT for BAO. Further prospective validation in larger cohorts is required before the score can inform treatment decisions.

## Introduction

Acute basilar artery occlusion (BAO) is a severe subtype of stroke that carries a dismal prognosis; without reperfusion, most patients die or survive with severe disabilities (1). Multiple observational studies and randomized clinical trials have demonstrated the superiority of endovascular therapy (EVT) over best medical treatment alone for BAO (2–6). This benefit was recently reinforced by VERITAS, an individual participant-data meta-analysis of the four randomized trials of EVT for BAO, which reported higher rates of favorable functional outcome and lower mortality with EVT than with medical management alone (7). Recently, the 2026 AHA/ASA stroke guidelines (8) issued a Class I recommendation for EVT in BAO presenting within 24 h of symptom onset with pc-ASPECTS of ≥6 and NIHSS of ≥10. Therefore, appropriate patient selection using baseline neuroimaging is important prior to EVT.

Pc-ASPECTS is a complex and subjective tool that may over- or under-estimate stroke severity depending on the structures involved (9, 10). For example, pc-ASPECTS assigns the same score to a patient with significant bilateral pontine infarction as to one with small bilateral cerebellar infarction, despite the former likely having a worse prognosis. Reported inter-rater agreement for pc-ASPECTS is only moderate, and its discriminative performance for functional outcome after EVT has varied across cohorts (9, 10).

Other posterior-circulation scoring systems have been developed, including the pons-midbrain index (PMI) and the Diffusion-Weighted Imaging Brainstem Score (DWI-BSS), derived from CT angiography source images (CTA-SI) and MRI DWI respectively; however, each has been validated in only a single study (11, 12).

Several further DWI-based systems have been proposed, including MRI pc-ASPECTS, the MRI-based score of Renard et al., the brainstem DWI lesion score, and a novel posterior circulation (PC) score (10, 13–15). Across these systems, reported discrimination for poor functional outcome has been moderate, but all require multi-component calculation with regional weighting, most were derived and validated in single cohorts, and inter-rater reliability is seldom reported.

Beyond direct measures of infarct extent, indirect imaging markers have been investigated as outcome predictors in posterior circulation stroke. Measures of global and regional brain atrophy have been associated with functional outcome after EVT for basilar occlusion and may capture baseline neuronal reserve that lesion-based scores do not (16, 17). Such markers are complementary to, rather than competing with, infarct-burden scores; they were not available in the present retrospective cohort. Taken together, existing posterior-circulation scores share three practical limitations: multi-region arithmetic that is difficult to perform under time pressure, equal weighting of structures with very unequal functional importance, and reliability that is infrequently reported.

We present the HALF-IT score, a novel binary posterior-circulation infarction score that focuses on the brainstem. This study investigated the utility of the HALF-IT score to predict functional outcomes of EVT in patients with BAO.

## Methods

### Design

We conducted a multicenter, retrospective cohort study at four comprehensive stroke centers between 2021 and 2025. The participating sites were HCA Houston Healthcare Kingwood, Kingwood, TX; Indiana University Health Methodist Hospital, Indianapolis, IN; the University of California, Los Angeles, Los Angeles, CA; and Bon Secours Mercy Health St. Vincent Medical Center, Toledo, OH. All are certified comprehensive stroke centers with 24-h neurointerventional coverage and continuous MRI availability. Annual thrombectomy volume ranged from approximately 60 to 200 procedures per site; site-level characteristics and MRI acquisition parameters are summarized in Supplementary Table S1.

Patients were eligible if they met all of the following inclusion criteria:

1. Age ≥18 years;
2. Acute basilar artery occlusion identified on CTA, MRA or digital subtraction angiography (DSA), with all patients ultimately undergoing DSA and attempted thrombectomy; and
3. A diagnostic brain MRI—including DWI, ADC, and FLAIR sequences—performed prior to DSA/thrombectomy.

Demographics, baseline clinical characteristics, periprocedural variables, and clinical outcomes up to 90 days were recorded. Stroke etiology was classified according to the TOAST criteria (18) on the basis of the completed diagnostic work-up. The level of basilar artery occlusion was determined on baseline CTA or MRA and confirmed on the pre-thrombectomy angiographic run, and was categorized as proximal (vertebrobasilar junction to the origin of the anterior inferior cerebellar artery), middle (anterior inferior cerebellar artery to the origin of the superior cerebellar artery), or distal (superior cerebellar artery origin to the basilar apex); patients in whom thrombus spanned more than one segment were classified by the most proximal segment involved. Reperfusion was graded with the modified Thrombolysis in Cerebral Infarction (mTICI) scale (19), the primary grading system used in the ATTENTION and BAOCHE trials (5, 6). Where intravenous thrombolysis was not administered, the documented reason was recorded. The study was determined to be exempt from IRB approval due to its retrospective design and the use of deidentified aggregate data.

### MRI acquisition

Baseline MRI was performed on GE (two sites) and Siemens (two sites) scanners at 1.5 T and 3.0 T according to each site's standard acute stroke protocol. Diffusion-weighted imaging was acquired as a single-shot spin-echo echo-planar sequence with *b* values of 0 and 1,000 s/mm^2^ at all sites, with one site additionally acquiring *b* = 500 s/mm^2^, and with automated ADC map reconstruction. Acquisition parameters varied slightly across sites and are provided in Supplementary Table S1. Because the HALF-IT score is a visual, proportion-based assessment rather than a volumetric measurement, it was applied identically across field strengths and protocols; the potential effect of slice thickness on assessment of small brainstem segments is addressed in the Limitations.

### The HALF-IT score

The HALF-IT score is a novel, simplified, dichotomized triaging tool focusing solely on the brainstem. A patient was classified as HALF-IT positive when a confluent acute infarct occupied 50% or more of the cross-sectional area of any single brainstem segment (midbrain, pons, or medulla) on the axial or coronal slice of maximal involvement, and HALF-IT negative when less than 50% of every segment was involved (Figure 1).

**Figure 1:**
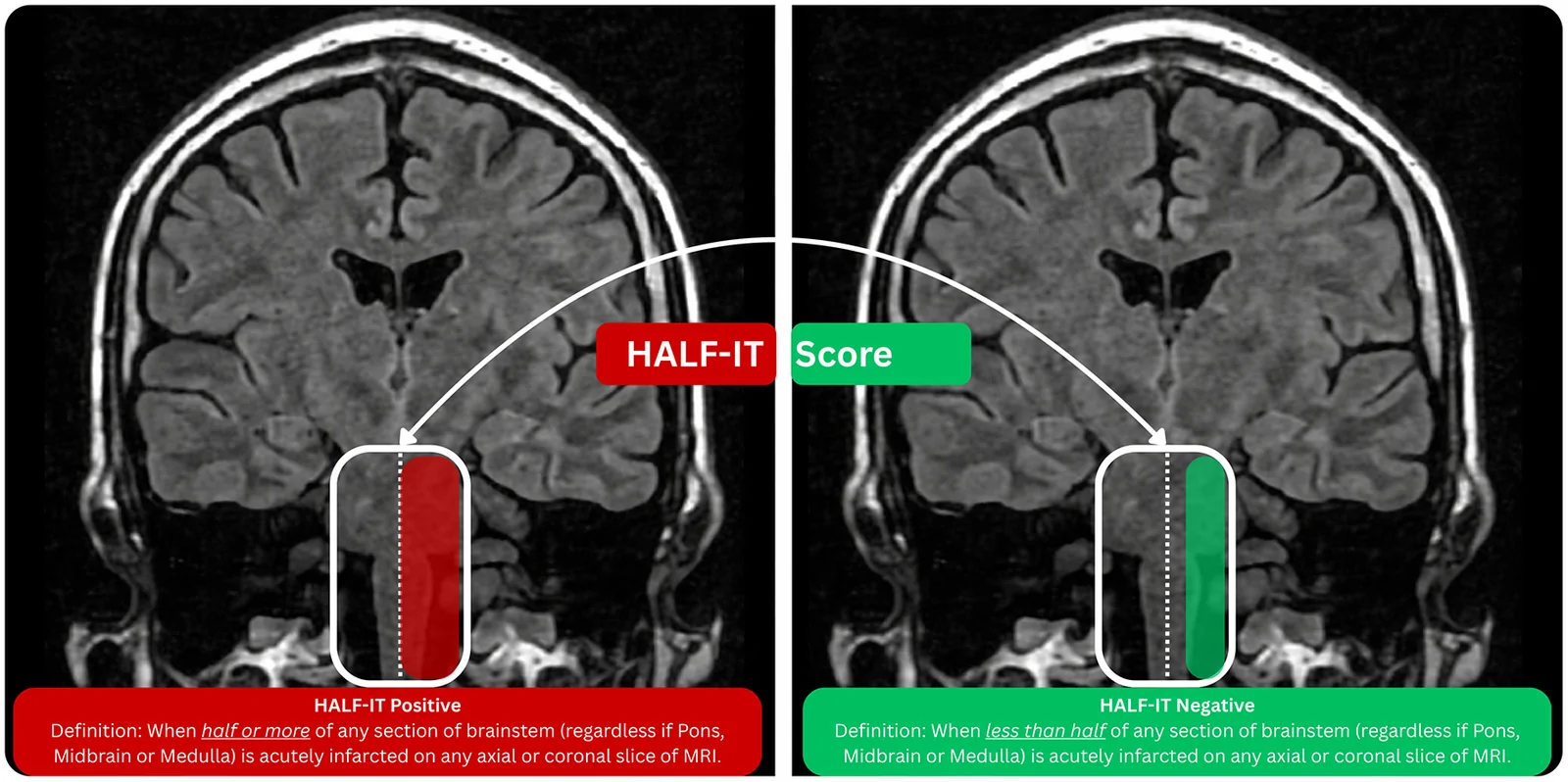
Visual representation of HALF-IT positive vs. HALF-IT negative.

Acute infarction was defined as unequivocal diffusion restriction (DWI hyperintensity with corresponding ADC hypointensity); FLAIR was used only to corroborate lesion extent and was not sufficient on its own to establish acuity. Because chronic infarction and small-vessel disease are FLAIR-hyperintense but lack diffusion restriction, they did not meet this definition. A scattered or punctate pattern of acute foci that did not coalesce into a confluent lesion occupying ≥50% of a segment was classified as HALF-IT negative.

The 50% threshold was pre-specified rather than derived from these data. It was chosen *a priori* because a ≥50% vs. < 50% cut-point is already used for regional grading within the pons-midbrain index and DWI-based brainstem scores (11, 12), and because a single dichotomous cut-point avoids the multi-region arithmetic that limits bedside use of existing scores. No alternative thresholds were tested, and the cut-point was not optimized against outcome data in this cohort. Involvement was assessed by visual inspection alone, without planimetry or volumetric segmentation, in order to reproduce the conditions of emergent decision-making. Baseline MRI sequences were independently reviewed by two neurologists; any disagreements were resolved by consensus with a board-certified endovascular neuroradiologist. Disagreement regarding the binary HALF-IT classification occurred in 1 of 37 cases (agreement 36/37, 97.3%; Cohen's κ = 0.94); that case was classified as HALF-IT negative by consensus.

### Analysis

All statistical analyses were performed using RStudio R 4.5.2. Baseline continuous variables were reported as median with interquartile range and compared using the Wilcoxon rank-sum test. Categorical variables were reported as counts and percentages and compared using the χ^2^ test or Fisher's exact test, or the Fisher–Freeman–Halton extension of Fisher's exact test for categorical variables with more than two levels. The primary efficacy outcome was a favorable functional outcome, defined as a modified Rankin Scale (mRS) score of 0–3 at 90 days. This threshold was selected because it was the primary endpoint of the ATTENTION and BAOCHE trials (5, 6) and is the accepted benchmark in basilar occlusion, where mRS 0–2 is achieved by a minority of survivors. Functional independence, defined as mRS 0–2, was analyzed and reported separately as a secondary outcome. Primary safety endpoints included symptomatic intracranial hemorrhage within 24 h post-procedure [SITS-MOST criteria (20)] and all-cause mortality at 90 days. Unadjusted associations between HALF-IT score and outcomes were evaluated using univariable logistic regression, with results reported as odds ratios (ORs) and 95% confidence intervals (CIs). Sensitivity analysis was performed on the primary efficacy outcome after excluding patients with failed reperfusion (mTICI < 2b). Ordinal logistic regression was used to evaluate mRS shift analyses. Multivariable logistic regression was performed to identify independent predictors of the primary efficacy outcome. Diagnostic performance metrics including sensitivity, specificity, positive predictive value (PPV), negative predictive value (NPV), and accuracy were compared among the three scores (HALF-IT, pc-ASPECTS, PMI) for predicting the primary efficacy outcome. Areas under the receiver-operating-characteristic curve (AUCs) were compared between HALF-IT and each of pc-ASPECTS and PMI using the DeLong test. Level of basilar occlusion and TOAST etiologic subtype were compared between the two HALF-IT groups. A sensitivity analysis of the primary efficacy outcome was performed with stratification by involvement of the proximal basilar segment, summarized with a Mantel–Haenszel common odds ratio and the Cochran–Mantel–Haenszel test.

## Results

### Baseline and procedure

37 patients (23 HALF-IT negative and 14 HALF-IT positive) met the inclusion criteria. Baseline characteristics, initial presentation, procedural details, angiographic outcomes, and discharge variables are presented in Table 1. In the HALF-IT positive group, the median age was 50 (32–58) years vs. 65 (58–74) years in the HALF-IT negative group (*p* = 0.001). HALF-IT positive patients presented with a higher median initial NIHSS [28 (22–30) vs. 7 (3–19), *p* = 0.001], higher median pons-midbrain index score [3.5 (2.8–5) vs 1 (0–2), *p* = 0.02], and lower median pc-ASPECTS score [6.5 (3.8–8) vs. 8 (6–9.5), *p* = 0.09] compared with the HALF-IT negative patients. None of the patients in the HALF-IT positive cohort received intravenous thrombolysis (IVT) vs. 26% in the HALF-IT negative group, but the difference was not statistically significant (*p* = 0.07).

**Table 1:** Baseline characteristics, initial presentation, procedural details, angiographic outcomes, and discharge variables.

Variable	HALF-IT positive (*N* = 14)	HALF-IT negative (*N* = 23)	*P* value^a^
Demographics and past medical history			
Age, median (IQR), year	50 (32–58)	65 (58–74)	0.001
Male, *n* (%)	10 (71.4%)	14 (60.9%)	0.72
Race, *n* (%)			
Caucasian/white	7 (50.0%)	13 (56.5%)	0.7
African American/black	5 (35.7%)	6 (26.0%)	0.7
Hispanic/latino	1 (7.0%)	2 (8.7%)	1
Other/unknown	1 (7.0%)	2 (8.7%)	1
Hypertension, *n* (%)	6 (42.9%)	17 (74.0%)	0.06
Diabetes mellitus, *n* (%)	4 (28.6%)	8 (34.8%)	1
Hyperlipidemia, *n* (%)	6 (42.9%)	13 (56.5%)	0.42
Atrial fibrillation, *n* (%)	1 (7.0%)	2 (8.7%)	1
Coronary artery disease/congestive heart failure, *n* (%)	3 (21.4%)	7 (30.4%)	0.7
Ejection fraction, median (IQR)	56 (40–60)	60 (53.3–62.8)	0.23
Previous stroke, *n* (%)	6 (42.9%)	8 (34.8%)	0.62
Smoker (current or former), *n* (%)	9 (64.3%)	11 (47.8%)	0.33
Baseline antiplatelet/anticoagulant use, *n* (%)	5 (35.7%)	9 (39.0%)	0.84
Premorbid mRS, *n* (%)			1
0–1	11 (78.6%)	19 (82.6%)	
≥2	3 (21.4%)	4 (17.4%)	
Initial presentation			
Onset to arrival, median (IQR), hours	8.8 (4.3–14)	7.7 (2.6–19.7)	0.88
Initial systolic blood pressure, median (IQR), mmHg	148 (129–166)	160 (139–189)	0.27
Initial NIHSS, median (IQR)	28 (22–30)	7 (3–19)	0.001
Intravenous thrombolysis (IVT) administration, n (%)	0 (0.0%)	6 (26.0%)	0.07
pc-ASPECTS, median (IQR)	6.5 (3.8–8)	8 (6–9.5)	0.09
Score ≤ 5, *n* (%)	5/12^b^ (41.7%)	5 (21.7%)	0.26
Pons-Midbrain Index, median (IQR)	3.5 (2.8–5)	1 (0–2)	0.02
Score ≥3, *n* (%)	9/12^b^ (75.0%)	5/19 (26.3%)	0.01
Stroke etiology (TOAST), *n* (%)			0.28
Large-artery atherosclerosis	3 (21.4%)	10 (43.5%)	
Cardioembolism	5 (35.7%)	6 (26.1%)	
Other determined	5 (35.7%)	3 (13.0%)	
Undetermined	1 (7.1%)	4 (17.4%)	
Level of basilar occlusion, *n* (%)			0.63
Proximal	6 (42.9%)	8 (34.8%)	
Middle	3 (21.4%)	9 (39.1%)	
Distal	5 (35.7%)	6 (26.1%)	
Vertebral artery V4 segment involvement, *n* (%)	6 (42.9%)	12 (52.2%)	0.58
Procedural details and angiographic outcomes			
Onset to puncture, median (IQR), hours	15.5 (10.7–30.6)	13 (6.5–42.2)	0.53
Arrival to puncture, median (IQR), hours	7.9 (3.5–18.3)	4.7 (2.4–10.7)	0.45
Onset to reperfusion, median (IQR), hours	16.9 (11.1–32)	15.8 (7.4–40.3)	0.65
Puncture to reperfusion, median (IQR), minutes	21 (18–46)	75 (36–100)	0.004
Anesthesia type, *n* (%)			0.28
General anesthesia	11 (78.6%)	13 (56.5%)	
Monitored anesthesia care	3 (21.4%)	10 (43.5%)	
First pass technique, *n* (%)			0.16
Aspiration only	10/13^c^ (77.0%)	11 (47.8%)	
Combined aspiration and stent-retriever	3/13^c^ (23.0%)	12 (52.2%)	
First pass effect, *n* (%)			
mTICI 2b-3	7/13^c^ (53.8%)	15 (65.2%)	0.5
mTICI 2c-3	6/13^c^ (46.2%)	15 (65.2%)	0.27
mTICI 3	5/13^c^ (38.5%)	12 (52.2%)	0.43
Final reperfusion, *n* (%)			
mTICI 2b-3	12 (85.7%)	23 (100.0%)	0.14
mTICI 2c-3	12 (85.7%)	21 (91.3%)	0.63
mTICI 3	10 (71.4%)	15 (65.2%)	1
Discharge variables			
Length of hospital stay, median (IQR), days	10.5 (5.8–21.3)	7 (6–11.5)	0.28
Discharge disposition, *n* (%)			
Home (± home health service)	2 (14.3%)	5 (21.7%)	1
Rehabilitation	3 (21.4%)	8 (34.8%)	0.47
Skilled nursing facility/long-term acute care	1 (7.0%)	2 (8.7%)	1
Hospice	1 (7.0%)	1 (4.4%)	1
Discharge mortality	7 (50.0%)	7 (30.4%)	0.23

^a^Calculated using the Wilcoxon rank-sum test for continuous variables and the chi-square/Fisher's exact test for categorical variables, or the Fisher–Freeman–Halton exact test where a variable had more than two levels. Patients whose thrombus spanned more than one basilar segment were classified by the most proximal segment involved. ^b^Baseline pc-ASPECTS and PMI scores available for only 12/14 HALF-IT positive patients. ^c^In one HALF-IT positive patient, intracranial access could not be achieved due to unfavorable vascular anatomy; first pass was therefore technically performed in 13/14 HALF-IT positive patients.

Although the HALF-IT positive patients had a significantly shorter puncture-to-final-reperfusion time compared with HALF-IT negative patients [21 (18–46) mins vs. 75 (36–100) mins, *p* = 0.004], the overall time from onset to reperfusion was similar between the two groups [16.9 (11.1–32) hrs vs. 15.8 (7.4–40.3) hrs, *p* = 0.65]. 23/23 (100%) of HALF-IT negative patients achieved final reperfusion of mTICI ≥2b compared with 12/14 (85.7%) of HALF-IT positive patients. In the latter cohort, one patient underwent DSA but EVT was aborted due to unfavorable vascular anatomy (final mTICI 0), and another achieved final mTICI 2a after 2 passes.

Stroke etiology and level of occlusion are summarized in Table 1. Final TOAST classification did not differ significantly between groups (*p* = 0.28). The level of basilar occlusion was also similarly distributed (*p* = 0.63): proximal 6/14 (42.9%) vs. 8/23 (34.8%), middle 3/14 (21.4%) vs. 9/23 (39.1%), and distal 5/14 (35.7%) vs. 6/23 (26.1%). Thrombus spanned more than one segment in 3/14 (21.4%) HALF-IT positive and 2/23 (8.7%) HALF-IT negative patients, and any involvement of the proximal segment was present in 6/14 (42.9%) and 8/23 (34.8%) respectively (*p* = 0.73). In the sensitivity analysis stratified by involvement of the proximal basilar segment, HALF-IT positivity remained associated with a lower rate of the primary efficacy outcome within both strata [proximal involvement: 1/6 (16.7%) vs. 5/8 (62.5%), *p* = 0.14; no proximal involvement: 1/8 (12.5%) vs. 10/14 (71.4%), *p* = 0.02; Mantel–Haenszel common OR 0.08, Cochran–Mantel–Haenszel *p* = 0.007]. Among the 14 HALF-IT positive patients, intravenous thrombolysis was withheld because of presentation beyond the thrombolysis window in 9, unknown or unwitnessed time of onset in 3, and a contraindication to thrombolysis in 2 (recent ischemic infarct within 3 months, and recent anterior cervical discectomy and fusion).

### Univariate regression

24-h /discharge and 90-day clinical outcomes are presented in Table 2. 90-day follow-up data were available for 14/14 (100%) of HALF-IT positive and 22/23 (95.7%) of HALF-IT negative patients. The 90-day mRS ordinal shift is presented in Figure 2. The rate of favorable functional outcome (mRS 0–3) was significantly lower in the HALF-IT positive compared with the HALF-IT negative group [2/14 (14.3%) vs. 15/22 (68.2%), OR 0.08 (0.01–0.45), *p* = 0.004]. Sensitivity analysis excluding the 2 HALF-IT positive patients with final mTICI < 2b also demonstrated a statistically significant difference in the primary efficacy outcome between the two groups [2/12 (16.7%) vs. 15/22 (68.2%), OR 0.09 (0.02–0.54), *p* = 0.01]. At 90 days, the rates of all-cause mortality (57% vs. 31.8%, *p* = 0.14) and mRS 5–6 (64.3% vs. 31.8%, *p* = 0.06) were numerically higher in the HALF-IT positive group but the difference did not reach statistical significance.

**Table 2:** 24-h /discharge and 90-day outcomes.

Variable	HALF-IT positive (*N* = 14)	HALF-IT negative (*N* = 23)	OR/cOR (95% CI)	*P* value^a^
Primary efficacy outcome				
90-day mRS 0–3, *n* (%)	2 (14.3%)	15/22^b^ (68.2%)	0.08 (0.01–0.45)	0.004
Secondary efficacy outcomes				
90-day mRS ordinal shift	—	—	cOR: 4.9 (1.3–18.5)	0.02
90-day mRS 0–2, *n* (%)	1 (7.0%)	11/22^b^ (50.0%)	0.08 (0.01–0.7)	0.02
24 hr/Discharge mRS ordinal shift	—	—	cOR: 4.2 (1.2–15)	0.03
24 hr/Discharge mRS 0–3, *n* (%)	1 (7.0%)	10 (43.5%)	0.1 (0.01–0.9)	0.04
24 hr/Discharge mRS 0–2, *n* (%)	1 (7.0%)	7 (30.4%)	0.18 (0.02–1.6)	0.12
24 hr/Discharge NIHSS, median (IQR)	35 (17.3–42)	4 (1–42)	—	0.03
90–day NIHSS, median (IQR)	42 (42–42)	3 (1–42)	—	0.056
Primary safety outcomes				
Symptomatic ICH (sICH) at 24 hr, *n* (%)	2 (14.3%)	3 (13.0%)	1.1 (0.2–7.6)	0.92
90-day mortality, *n* (%)	8 (57.0%)	7/22 (31.8%)	2.9 (0.7–11.4)	0.14
Secondary safety outcomes				
Any hemorrhage at 24 hr, *n* (%)	5 (35.7%)	7 (30.4%)	1.3 (0.3–5.2)	0.74
24 hr/Discharge mortality, *n* (%)	7 (50.0%)	7 (30.4%)	2.3 (0.6–9)	0.24
90-day mRS 5–6, *n* (%)	9 (64.3%)	7/22 (31.8%)	3.9 (0.9–15.9)	0.06

CI, confidence interval; cOR, common odds ratio; OR, odds ratio. ^a^Calculated using proportional ordinal logistic regression with common odds ratio for mRS ordinal shift, and univariate regression for the remainder. ^b^90-day follow-up data were available for 14/14 of HALF-IT positive and 22/23 of HALF-IT negative patients.

**Figure 2:**
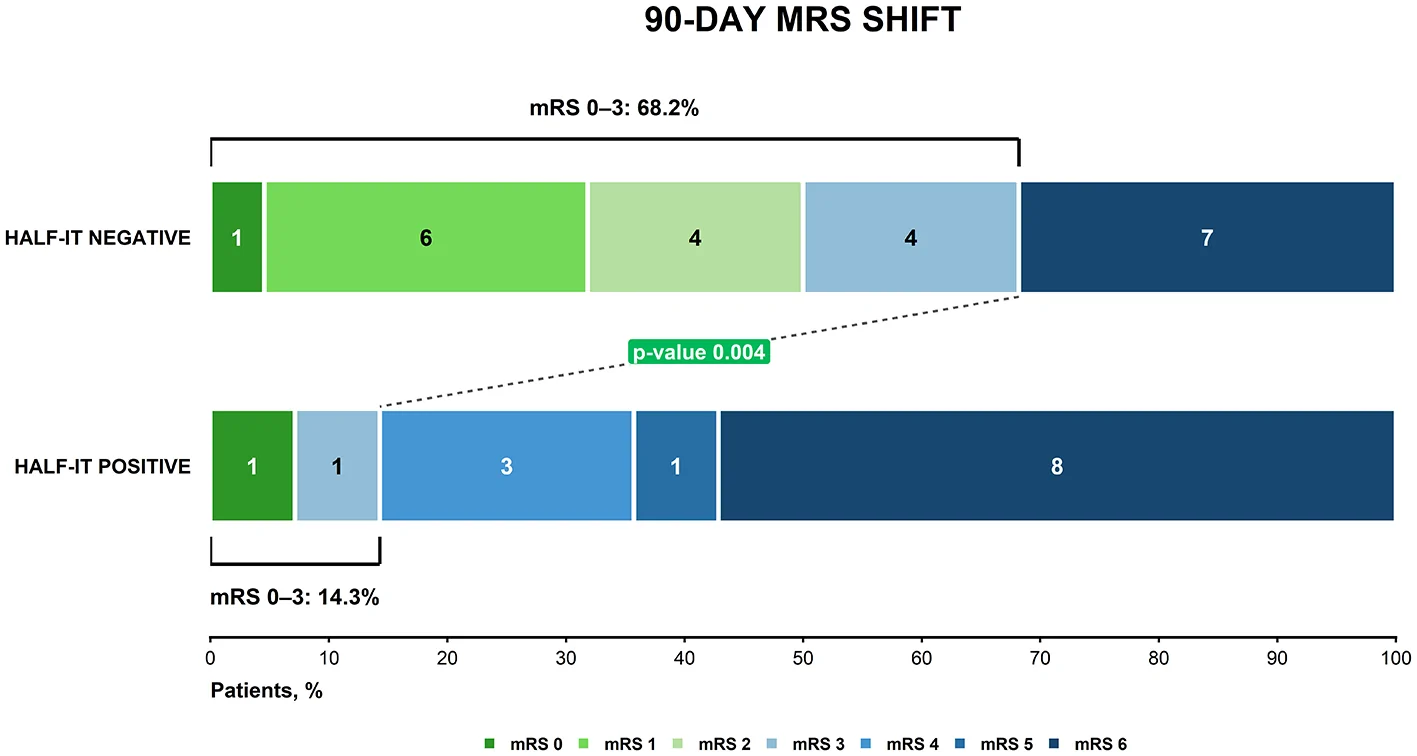
90-day mRS shift. The HALF-IT positive group had a significantly lower rate of favorable functional outcome (mRS 0–3) at 90 days compared with the HALF-IT negative group (14.3% vs. 68.2%, *p* = 0.004).

### Multivariate regression

Multivariate logistic regression (Table 3) was performed to identify independent predictors of the primary efficacy outcome. HALF-IT positive status was independently associated with a lower rate of mRS 0–3 at 90 days even after adjusting for age, initial NIHSS, onset-to-reperfusion time, and rate of complete reperfusion (final mTICI 3) [adjusted OR 0.001, 95% CI 0–0.55, *p* = 0.03]. Intravenous thrombolysis (IVT) administration was excluded from the primary model due to complete separation, since all patients who received IVT in the HALF-IT negative group achieved the primary efficacy endpoint. A sensitivity analysis using Firth penalized regression, which is more stable under separation, yielded a consistent direction and significance of association after including IVT [adjusted OR 0.02, 95% CI 0–0.84, *p* = 0.02]. TOAST etiologic subtype and level of basilar occlusion were not entered into the adjusted models: with limited sample, a model already containing five covariates cannot reliably accommodate additional variables, and both were similarly distributed between the HALF-IT groups (Table 1). Notably, baseline pc-ASPECTS and pons-midbrain index scores were not included in the model due to potential multicollinearity with the HALF-IT score.

**Table 3:** Multivariate regression of 90-day mRS 0–3.

Predictor	Adjusted odds ratio (aOR)	95% confidence interval	*P* value
Primary model (excluding IVT administration)			
HALF-IT positive status	0.001	0–0.55	0.03
Age	0.86	0.75–0.98	0.02
Initial NIHSS	0.93	0.83–1.04	0.21
Onset-to-reperfusion time	1	1–1	0.19
Final reperfusion mTICI 3	12.47	0.52–299	0.12
Sensitivity analysis/firth penalized regression (including IVT administration)			
HALF-IT positive status	0.02	0–0.84	0.02
Age	0.91	0.84–0.99	0.01
Initial NIHSS	0.95	0.87–1.05	0.35
IVT administration	5.5	0.31–96	0.22
Onset-to-reperfusion time	1	1–1	0.47
Final reperfusion mTICI 3	5	0.62–40	0.13

IVT, intravenous thrombolysis. IVT administration was excluded from the primary model due to complete separation; a sensitivity analysis was performed using Firth penalized regression to account for separation.

### Comparison with pc-ASPECTS and PMI

Furthermore, we compared the predictive performance of the HALF-IT score with pc-ASPECTS ( ≤ 5 vs. >5) and PMI (< 3 vs. ≥3) within our study population. Among the three scores, HALF-IT had numerically the highest sensitivity, specificity, positive predictive value, negative predictive value, and accuracy (Figure 3b). On ROC analysis (Figure 3a), the AUC was 0.786 for HALF-IT, 0.75 for pc-ASPECTS, and 0.643 for PMI; differences in discrimination between HALF-IT and the comparator scores were assessed using the DeLong test (HALF-IT vs. pc-ASPECTS, p= 0.75; HALF-IT vs. PMI, *p* = 0.15). Neither comparison reached statistical significance; the present data therefore do not demonstrate that HALF-IT discriminates better than pc-ASPECTS or PMI. Given the sample size, the confidence intervals around all three AUCs are wide and overlapping, and the study was not powered for a head-to-head comparison of discrimination.

**Figure 3:**
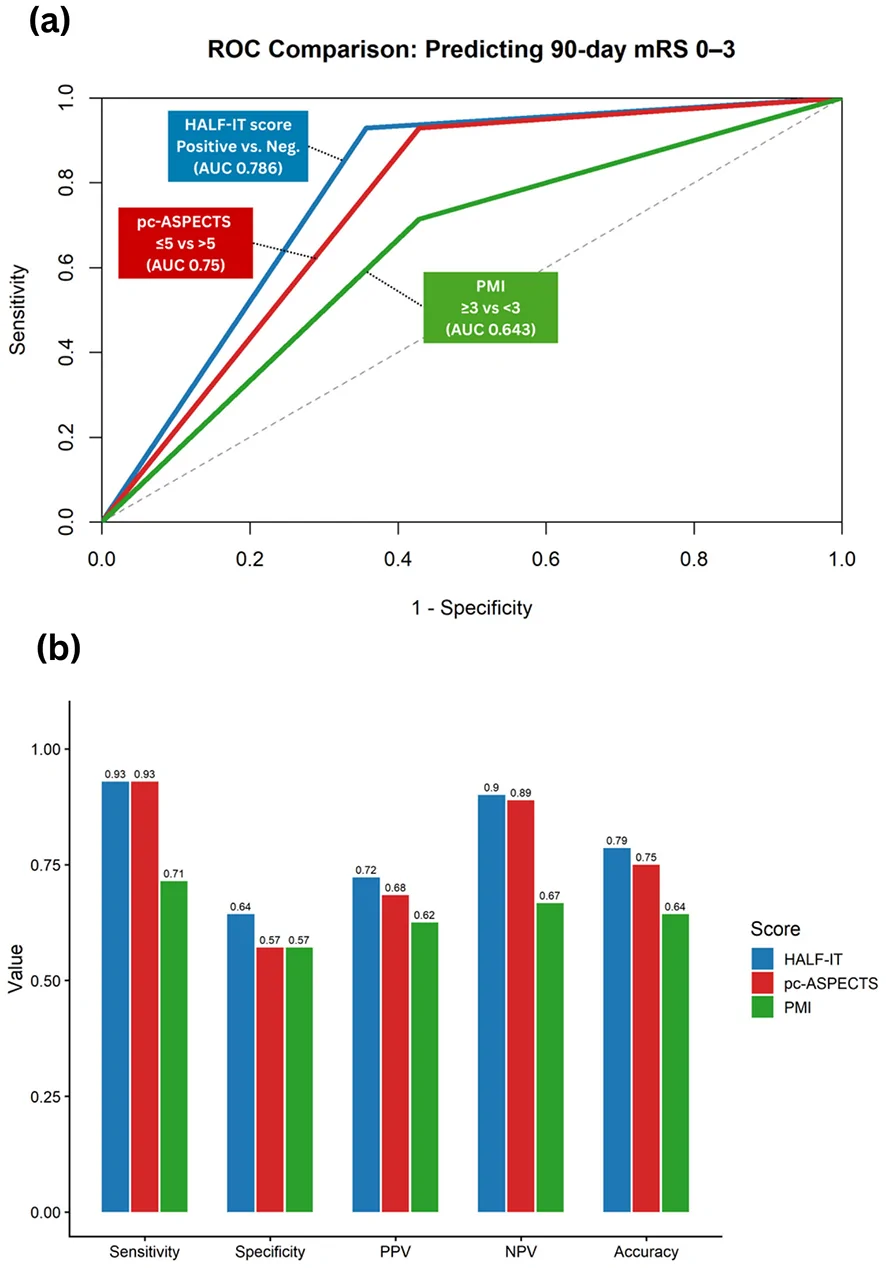
Comparison of the performance of HALF-IT, pc-ASPECTS, and PMI scores in predicting the rate of 90-day mRS 0–3. **(a)** Receiver operating characteristics (ROC) curves comparing areas under curve (AUC) among the three scores. **(b)** Comparison of diagnostic performance metrics. NPV, negative predictive value; PPV, positive predictive value.

## Discussion

In this multicenter retrospective cohort study of patients with acute BAO undergoing EVT, the HALF-IT score—a binary, MRI-based measure of brainstem infarct burden—was independently associated with 90-day functional outcome. Patients classified as HALF-IT positive demonstrated a substantially lower rate of favorable functional outcome (mRS 0–3) at 90 days, and this association remained significant after adjusting for age, initial NIHSS, onset-to-reperfusion time, and rate of complete reperfusion (final mTICI 3); intravenous thrombolysis was accommodated in a Firth penalized sensitivity model because of complete separation. These associations should, however, be interpreted in light of substantial baseline differences between the two groups.

A notable observation was that HALF-IT positive patients had significantly shorter puncture-to-reperfusion time yet experienced worse functional outcomes, despite similar rates of successful final reperfusion. One interpretation is that baseline brainstem tissue injury, rather than procedural factors, was an important determinant of clinical outcome. We present this as a provisional interpretation rather than a demonstrated mechanism, because the two groups differed markedly at baseline and unmeasured confounding cannot be excluded. This is consistent with futile recanalization, in which restoration of vessel patency does not translate into clinical benefit because the infarcted tissue is no longer salvageable. The shorter procedure times most likely reflect the nature of the underlying occlusion in high-burden cases—a proximal, aspiration-amenable clot frequently retrieved in fewer passes, as suggested by the higher proportion of aspiration-only first-pass attempts in this group (77.0% vs. 47.8%)—and, earlier procedural termination in 2 patients. This observation is consistent with, though not proof of, the premise that HALF-IT reflects irreversible baseline injury; the persistence of worse outcomes in the sensitivity analysis restricted to patients with successful reperfusion (mTICI ≥2b) points in the same direction.

Several baseline differences deserve emphasis. HALF-IT positive patients were younger, presented with a markedly higher NIHSS (median 28 vs. 7), and none received intravenous thrombolysis. A higher presenting NIHSS is itself among the strongest predictors of poor outcome in basilar occlusion, and HALF-IT positivity may in part be a radiological expression of the same underlying severity rather than an independent construct. An alternative and important explanation is that the two groups differed in the anatomy of the occlusion: proximal basilar occlusions are more often atherosclerotic, are associated with a larger pontine infarct burden, and carry a worse prognosis than distal or top-of-the-basilar occlusions, which more often produce thalamic and occipital rather than brainstem injury. It is therefore plausible that HALF-IT positivity partly identifies proximal basilar occlusion rather than tissue injury *per se*. In this cohort, however, occlusion level was distributed similarly in the two groups (proximal 42.9% vs. 34.8%, middle 21.4% vs. 39.1%, distal 35.7% vs. 26.1%; *p* = 0.63), as was TOAST etiologic subtype (*p* = 0.28), so occlusion topography does not appear to account for the difference in outcome we observed. The association also persisted within strata defined by involvement of the proximal basilar segment.

These findings are clinically relevant because appropriate patient selection using baseline neuroimaging is important prior to EVT in BAO. The 2026 AHA/ASA guidelines (8) recognized the lower sensitivity of plain CT compared with CTP, MRI DWI/FLAIR, or MR DWI-PWI for selecting posterior-circulation strokes for EVT. Previous trials relied mainly on pc-ASPECTS, which assigns equal weight to brainstem, cerebellar, thalamic, and occipital lesions, potentially underestimating the disproportionate functional impact of brainstem infarction. As noted before, anatomically distinct patterns with very different functional consequences can share an identical pc-ASPECTS value.

HALF-IT was developed to address this limitation by focusing exclusively on the brainstem. Moreover, in contrast to existing posterior circulation infarction scores (Table 4) (9–15), we prioritized simplicity over granularity. Whereas previous scores require multi-component calculations and regional weighting, HALF-IT reduces interpretation to a quick binary visual assessment: ≥50% of brainstem involvement on any axial or coronal slice of pre-treatment MRI is considered HALF-IT positive whereas < 50% translates to HALF-IT negative. The simplicity of this approach may reduce inter-rater variability and expedite decision-making in emergent settings.

**Table 4:** Summary of existing posterior circulation infarction scores.

Study	Score	Required imaging modality	Score range	Score components
Puetz et al. (9)	pc-ASPECTS	Non-contrast CT or CTA source images (CTA-SI)	0–10 (points subtracted from 10)	Midbrain: 2 points
				Pons: 2 points
				Cerebellar hemispheres: 1 point each
				Occipital lobes: 1 point each
				Thalami: 1 point each
Schaefer et al. (11)	Pons-Midbrain Index (PMI)	CTA source images (CTA-SI)	0–8 (points added to zero)	Midbrain (each half separately): 0 (no hypoattenuation); 1 point (< 50% hypoattenuation); 2 points (>50% hypoattenuation)
				Pons (each half separately): 0 (no hypoattenuation); 1 point (< 50% hypoattenuation); 2 points (>50% hypoattenuation)
Mourand et al. (12)	DWI Brainstem Score (BSS)	MRI DWI	0–12 (points added to zero)	Midbrain (each half separately): 0 (no hypoattenuation); 1 point (< 50% hypoattenuation); 2 points (>50% hypoattenuation)
				Pons (each half separately): 0 (no hypoattenuation); 1 point (< 50% hypoattenuation); 2 points (>50% hypoattenuation)
				Medulla (each half separately): 0 (no hypoattenuation); 1 point (< 50% hypoattenuation); 2 points (>50% hypoattenuation)
Tei et al. (10)	MRI pc-ASPECTS	MRI DWI	0–10 (points subtracted from 10)	Same as the original pc-ASPECTS score.
Renard et al. (13)	—	MRI DWI	0–10 (points added to zero)	Midbrain: 1 point (unilateral); 2 points (bilateral)
				Pons: 1 point (unilateral); 2 points (bilateral)
				Medulla: 1 point (unilateral); 2 points (bilateral)
				Cerebellum: 1 point (unilateral); 2 points (bilateral)
				Temporo-occipital lobe: 0.5 points (unilateral); 1 point (bilateral)
				Thalamus: 0.5 points (unilateral); 1 point (bilateral)
Cho et al. (14)	Brainstem DWI Lesion Score	MRI DWI	0–22 (points added to zero)	Midbrain: 0–8 affected arterial territories (4 on each side)
				Pons: 0–6 affected arterial territories (3 on each side)
				Medulla: 0–8 affected arterial territories (4 on each side)
Bai et al. (15)	Novel posterior circulation (PC) score	MRI DWI	0–27 (points added to zero)	Midbrain, Pons, & Medulla (Left, Right, and Midline regions separately): 0 (no high signal); 1, lesion area < 50% of each half; and 2, ≥50% high signal of each half. Midline involved = Additional 1 score; total 0–15
				Cerebellum, Occipital lobes, & Thalami (each half separately): 0 (no high signal); 1, (lesion area < 50%); and 2, (≥50% high signal); total 0–12

Practically, the HALF-IT score may identify a subgroup of patients with basilar artery occlusion who have a higher baseline brainstem infarct burden and a potentially lower likelihood of meaningful functional recovery despite successful angiographic reperfusion. However, because this is a proof-of-concept study, we are not proposing that thrombectomy should be withheld on the basis of HALF-IT positivity alone. Rather, the HALF-IT score can help predict outcomes and inform goals-of-care discussions regarding prognosis with patients and families. Conceptually, HALF-IT positive patients may represent the posterior-circulation equivalent of large-core infarction, in whom the benefit of EVT remains uncertain.

Our study has several limitations. First, this was a retrospective study with a modest sample size (37 patients). Although effect sizes were statistically significant, several estimates had wide confidence intervals; therefore, the true magnitude of the association between HALF-IT score and clinical outcomes should be interpreted with caution at this hypothesis-generating stage. Second, baseline images were not evaluated by an independent core lab. Although interrater agreement was favorable with disagreement in only one case, none of the readers were blinded to the study which can introduce measurement bias. Third, although MRI remains the gold standard for acute infarct detection and infarct burden estimation, obtaining baseline MRI in routine practice may introduce workflow delays and limit generalizability, particularly in centers that rely primarily on non-contrast CT for decision-making. Fourth, this study did not compare the outcomes of HALF-IT positive patients who underwent thrombectomy with those of HALF-IT positive patients who did not, as essentially all included patients underwent EVT. The HALF-IT score therefore compares the outcomes of thrombectomy between HALF-IT positive and HALF-IT negative patients, rather than testing whether thrombectomy itself is beneficial in HALF-IT positive patients. Whether such patients benefit from EVT would require a dedicated comparison with an untreated group. Future studies should also evaluate whether the HALF-IT score can be reliably reproduced using non-contrast CT or CT perfusion, which may enhance its pragmatic applicability. Fifth, the two groups differed substantially in age, presenting NIHSS, and intravenous thrombolysis exposure, and residual confounding cannot be excluded despite multivariable adjustment. Fianlly, although HALF-IT showed numerically higher discrimination than pc-ASPECTS and PMI, the DeLong comparisons were not statistically significant, so this study is not evidence that HALF-IT outperforms existing scores; establishing superiority would require a substantially larger, adequately powered cohort. Further prospective validation in larger, multicenter cohorts is warranted to confirm the predictive value, reproducibility, and clinical utility of the HALF-IT score.

## Conclusion

In this small, retrospective, multicenter cohort, HALF-IT positivity was associated with worse 90-day functional outcomes after EVT for basilar artery occlusion and may help identify patients with a higher baseline brainstem infarct burden. These findings are hypothesis-generating: the HALF-IT positive and negative groups differed substantially at baseline, the score did not demonstrate statistically superior discrimination compared with pc-ASPECTS, and the sample was too small to exclude residual confounding by clinical severity, stroke etiology, or level of occlusion. Prospective validation in larger, adequately powered cohorts—including formal assessment of inter-rater reliability and head-to-head comparison with existing scores—is needed before the HALF-IT score can be used to inform treatment or prognostic decisions.

## Data Availability

The original contributions presented in the study are included in the article/Supplementary material, further inquiries can be directed to the corresponding author.
