# Supplementary material for: The HALF-IT score: proposing a simplified mechanical thrombectomy triage tool for basilar artery occlusion
Source: Front Neurol. 2026 Oct 14;17:1915436. doi: 10.3389/fneur.2026.1915436 (PMC13713328; doi:10.3389/fneur.2026.1915436)
Supplement: Supplementary file 1 [file Table_1.docx]

**Supplementary Table S1. Characteristics of the participating centers and site-level MRI acquisition parameters.**

| **Site** | **Annual thrombectomy volume** | **MRI vendor** | **Field strength** | **DWI b values (s/mm²)** | **Slice thickness (mm)** | **In-plane pixel spacing (mm)** |
| --- | --- | --- | --- | --- | --- | --- |
| 1. HCA Houston Healthcare Kingwood, Kingwood, TX | 100–120 | GE | 1.5 T | 0, 1000 | 5.0 | 0.94 × 0.94 |
| 2. Indiana University Health Methodist Hospital, Indianapolis, IN | 150–200 | Siemens | 1.5 T | 0, 1000 | 2.0 | 1.00 × 1.00 |
| 3. University of California, Los Angeles, Los Angeles, CA | 60 | Siemens | 1.5 T and 3.0 T | 0, 500, 1000 | 5.0 | 1.70 x 1.70 (1.5 T); 1.10 x 1.10 (3 T) |
| 4. Bon Secours Mercy Health St. Vincent Medical Center, Toledo, OH | 80–90 | GE | 1.5 T and 3.0 T | 0, 1000 | 5.0 | 1.00 × 1.00 |

All four centers are certified comprehensive stroke centers with 24-hour neurointerventional coverage and continuous MRI availability. Annual thrombectomy volume refers to all mechanical thrombectomy procedures performed at the site, anterior and posterior circulation combined.

DWI, diffusion-weighted imaging.

Diffusion-weighted imaging was acquired as a single-shot spin-echo echo-planar sequence with automated ADC map reconstruction at all sites.
